# Low folate intake and serum levels are associated with higher body mass index and abdominal fat accumulation: a case control study

**DOI:** 10.1186/s12937-020-00572-6

**Published:** 2020-06-04

**Authors:** Monika A. Mlodzik-Czyzewska, Anna M. Malinowska, Agata Chmurzynska

**Affiliations:** Institute of Human Nutrition and Dietetics, Poznań University of Life Sciences, Wojska Polskiego 31, 60-624 Poznań, Poland

**Keywords:** Folate, Body weight, Body fat, *MTHFR*, *DHFR*

## Abstract

**Background:**

The link between folate metabolism and obesity has recently been underlined, suggesting that folate deficiency may lead to body weight gain and adiposity. We thus wished to determine whether the inefficiency in folate metabolism caused by genetic variation in the *MTHFR* and *DHFR* genes in folate metabolism, or inadequate folate intake, is associated with obesity.

**Methods:**

A case–control study including 421 healthy participants (aged 20–40) was performed in Poznań, Poland. The cases were 213 subjects with BMI > 25 kg/m^2^, while the controls were 208 subjects with BMI < 25 kg/m^2^. Genotyping of rs70991108 (*DHFR*) and rs1801133 (*MTHFR*) was performed using TaqMan probes. Serum folate concentrations were measured using an enzyme-linked immunosorbent assay and homocysteine was assessed with high performance liquid chromatography.

**Results:**

Subjects with overweight and obesity had 12% lower folate intake (*p* < 0.05) and 8.5% lower folate serum concentrations (*p* < 0.01) than the controls. Serum folate concentrations and folate intake were inversely associated with body fat percentage (*p* < 0.05) and waist circumference (*p* < 0.05 and *p* < 0.001, respectively). Serum folate concentration, though not folate intake, was negatively associated with WHR and BMI (*p* < 0.05, for both associations).

**Conclusions:**

Lower folate intake and serum levels are weakly, but independently, associated with greater body weight and central adiposity in people aged 20–40. *MTHFR* and *DHFR* polymorphism seems not to have significant impact on body weight.

## Introduction

Although the relationship between excess calorie intake and obesity is recognized, the effect of micronutrient status on body mass determination is still unclear [1]. Previous research has revealed that individuals with obesity tend to have low intakes of vitamins and are more predisposed to deficiencies of vitamins A, B, D, E, and K [2–4]. Several studies have also suggested that micronutrient deficiency, especially of vitamin B_9_ (folate), can affect lipid [5] and energy metabolism [6].

The biologically active form of folate is tetrahydrofolate (THF), which acts as a coenzyme in folate-dependent reactions, such as biosynthesis of certain amino acids, purine bases, and thymine [7]. Deregulation of folate metabolism is associated with diverse metabolic alterations, including insulin resistance [8], metabolic syndrome [9], fatty liver disease [10] and lipoprotein profile imbalance [11]. Furthermore, folate deficiency may lead to body weight gain and adiposity [12]. Several studies has indicated a link between low folate intake or low serum folate concentration and greater body mass, BMI, overall fat accumulation, and higher waist circumference [13–16]. Yet a recent meta-analysis of 16 studies measuring folate concentration and BMI found no association between folate level and BMI [17], leaving open the question of whether this association exists. Although the link between obesity and folate status has gained much attention, the role of folate in obesity has not yet been explained. Recent studies have suggested that obesity may be another factor impairing folate metabolism and affecting its requirements [18]. For instance, Ortega et al. [19] showed that women with obesity with similar folate intake to nonobese women had lower serum folate level.

Given all this information, we sought here to test the hypothesis that inefficiency in folate metabolism caused by genetic variation in key genes in folate metabolism or inadequate folate intake is associated with greater body weight and excess accumulation of body fat. We selected only functional SNP polymorphisms such as rs70991108 (*DHFR*) and rs1801133 (*MTHFR)*, which have known mechanisms of action. We simultaneously took into account dietary intake of folate, folate and homocysteine (Hcy) concentrations, and the genotypes of *MTHFR* and *DHFR* in order to give us a comprehensive look at the associations between folate metabolism and obesity. Overall, we investigated the associations between: 1) folate intake and genotype and serum concentrations of folate and Hcy 2) folate intake, serum folate concentrations, and genotype and anthropometric parameters.

## Methods

### Design and participants

**Fig. 1:**
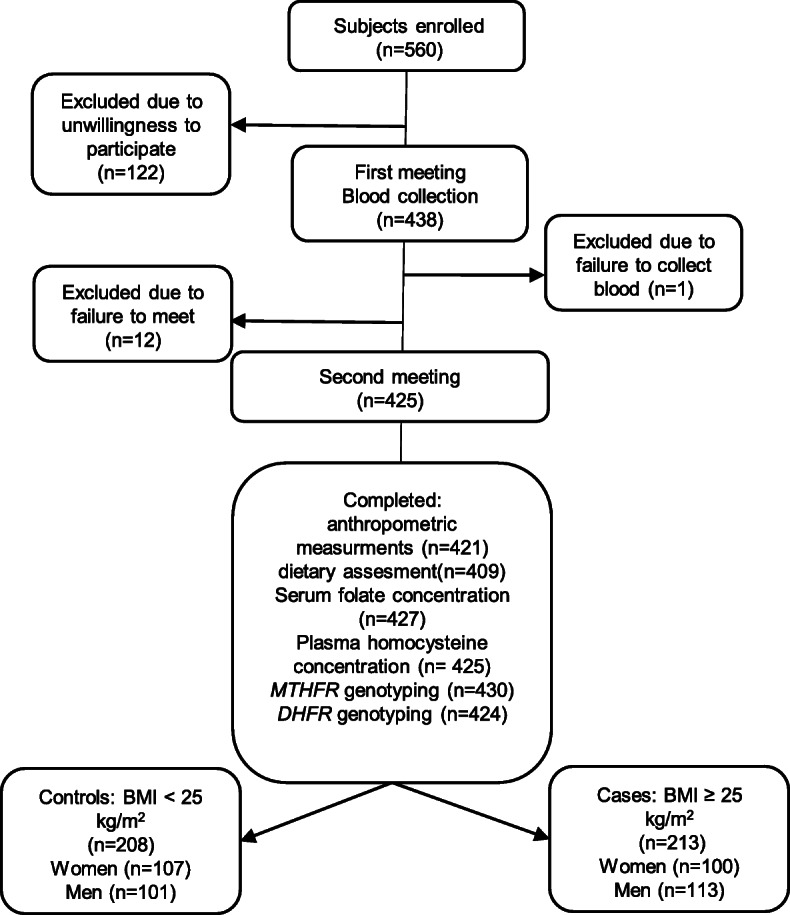
Flow of the study

Participants were enrolled for a case-control study [10]. The cases were 213 subjects with BMI > 25 kg/m^2^, while the controls were 208 subjects with BMI < 25 kg/m^2^. Recruitment was conducted in Poznań, Poland, using online advertisements, paper leaflets and taking advantage of the snowball sampling technique. Four hundred and twenty-one healthy adult subjects were recruited in spring and autumn in 2016–2018 and met the inclusion criteria, which were that they were of Caucasian race and aged 20 to 40. Because participants were originally enrolled for the study on fat taste and preference and intake of high-fat food, the exclusion criteria were pregnancy or lactation; chronic diseases like cancer, diabetes, and hyperthyroidism; use of medications known to affect taste, body weight, lipid profile, or appetite moderate or heavy smoking (more than one pack per week); recent dieting; or weight change of more than 5 kg in the past 3 months. The research protocol was approved by the Local Ethics Committee (number 966/2015). The flow of the study is presented in Fig. 1.

### Dietary assessment

Food intake was assessed using three-day food records. Participants were asked to report all food beverages and supplements consumed over 3 days (constituting 2 week days and one weekend day) in a food diary, after receiving individual training. The mean daily intake of total energy and the mean folate intake (dietary folate equivalent; DFE) were estimated using the computer software package Diet 6.0 (National Food and Nutrition Institute, Poland). Dietary pattern was assessment using an a priori approach, which included the Healthy Diet Indicator (HDI) [20]. To identify misreporters (underreporters and overreporters), the Goldberg and Black cutoff method was used. This procedure have been described in details in Malinowska et al. [20].

### Anthropometric measurements

The following anthropometric measurements were made: body height and weight, body fat percentage, and waist and hip circumferences. Height was measured to the nearest 0.5 cm using a WPT 100/200 OW stadiometer (RadWag, Poznań, Poland). Weight was measured to the nearest 0.01 kg following an overnight fast, using a calibrated scale included in the Bod Pod (Cosmed, Rome, Italy). BMI was calculated as body weight in kilograms divided by height in meters squared. Fat mass and lean body mass were determined using whole-body air-displacement plethysmography (BodPod). Subjects were tested while wearing only tight-fitting compression shorts or swimsuit and swim cap. Hip and waist circumferences were measured using nonelastic tape to 0.5 cm. Waist circumference was measured at the midpoint between the lowest rib and the top of the iliac crest. All measurements were performed by the same evaluator.

### Biochemical analysis

Blood samples for the biochemical measurements were allowed to clot at room temperature for 30 min. Serum was separated by centrifugation and stored at − 80 °C until analysis was performed. Serum folate concentrations were estimated using an enzyme-linked immunosorbent assay method (Folic Acid/Vitamin B9 ELISA Kit, Elabscience), following the manufacturer’s directions. Total Hcy concentrations were measured in plasma samples after derivatization using high performance liquid chromatography (HPLC) with UV detection [21].

### Genotyping

DNA was isolated from fresh blood collected in EDTA tubes, using a NucleoSpin Blood kit (Macherey-Nagel, Germany). Genotyping of rs70991108 in *DHFR* gene and rs1801133 in *MTHFR* gene was performed using TaqMan probes (single tube assays, Thermo Scientific) on a LightCycler 480 instrument (Roche Diagnostics, Switzerland).

### Statistical analysis

Crude analyses of the differences between the BMI subgroups were examined using Student’s *t*-test. To determine the associations between continuous variables, we then used multiple linear regression. The models for BMI, body fat percentage, waist and hip circumferences, and WHR were adjusted for diet quality (HDI), physical activity, sex, total energy intake, *MTHFR* and *DHFR* polymorphisms, and misreporting. To analyze the associations between folate metabolism and anthropometric parameters, we used multiple linear regression with adjustments for total energy intake, HDI, sex, physical activity, and misreporting. Logistic regression was used to calculate the odds ratio (OR) of being overweight or obese. Only the dominant inheritance model was tested. Due to much lower minor allele frequency and knowing that the minor allele has a causative effect on phenotype, we compared two genotype groups (CC vs. CT + TT in the *MTHFR* gene and 19 bp (−/−) vs. 19 bp (+/+) + 19 bp (+/−) in the *DHFR* gene). Data were analyzed using Statistica software (StatSoft, Tulsa, OK, USA).

## Results

### Population characteristics

The characteristics of the group are presented in Table 1. Of the 421 participants, 207 were women and 214 men. In the total population, the mean age was 27.6 years, the mean daily energy intake was 2152 kcal, the mean folate intake was 340 μg DFE/day, the mean serum folate concentration was 36.5 ng/ml, and the mean plasma Hcy concentration was 10.7 μM.

**Table 1:** Characteristics of the study group

Parameters	Mean ± SD (95%CI)
Age [years]	27.5 ± 5.5 (27.9; 28.0)
Men/women	207/214
Body mass [kg]	78.6 ± 18.1 (76.8; 80.3)
FM [%]	29.2 ± 10.8 (28.2; 30.2)
Waist circumference [cm]	83.9 ± 13.5 (82.6; 85.2)
Hip circumference [cm]	102 ± 9 (101; 103)
BMI [kg/m^2^]	26.0 ± 5.3 (25.4; 26.5)
WHR	0.84 ± 0.36 (0.80; 0.87)
Total calorie intake [kcal/day]	2152 ± 654 (2090; 2215)
DFE intake [μg/day]	340 ± 196 (320; 358)
% energy from fat	33.4 ± 7.3 (32.7; 34.1)
% energy from protein	16.5 ± 3.7 (16.1; 16.9)
% energy from carbohydrates	45.9 ± 7.4 (45.2; 46.7)
Serum folate concentration [ng/ml]	36.5 ± 12.4 (35.3; 37.6)
Plasma Hcy concentration [μmol/L]	10.7 ± 3.31 (10.4; 11.1)
*MTHFR* frequencies	Genotype: CC, 0.44; CT, 0.44; TT, 0.12 Allele C, 0.88; T, 0.55
*DHFR* frequencies	Genotype 19 bp (−/−), 0.30; 19 bp (+/−), 0.46; 19 bp (+/+) 0.24 Allele 19 bp (−), 0.76, 19 bp (+), 0.70

*Abbreviations*: *BMI* Body mass index, *DFE* Dietary folate equivalent, *FM* Fat mass, *WHR* Waist-to-hip ratio;

### Factors affecting folate and Hcy concentrations

First we examined the factors which could affect serum folate and Hcy concentrations. Serum folate concentrations were associated with HDI and *MTHFR* polymorphism (*p* < 0.01 for both associations), whereas the Hcy concentration was associated with sex (*p* < 0.001); see Table 2. Specifically, the T allele carriers of *MTHFR* had 11% higher serum folate concentration than individuals with the CC genotype, whereas women had 16% lower Hcy concentrations than men.

**Table 2:** Determinants of serum folate and homocysteine concentrations in people aged 20–40 years; *n* = 421

	Serum folate concentration [ng/ml]		Plasma Hcy concentration [μmol/L]	
	ß	*p*	ß	*p*
Folate intake [μ/day]	0.039	NS	−0.102	NS
HDI	**0.147**	**< 0.01**	0.06	NS
Sex, male	−0.036	NS	**0.301**	**< 0.001**
*DHFR*, major allele	−0.018	NS	−0.092	NS
*MTHFR*, major allele	**−0.145**	**< 0.01**	− 0.016	NS

*P* values < 0.05 were considered significant

The regression models were adjusted for total calorie intake, misreporting, and physical activity

*Abbreviations*: *NS* Not significant at *p* > 0.05, *HDI* Healthy Diet Indicator, *Hcy* Homocysteine

### Folate metabolism and obesity

To determine the differences in folate metabolism between cases and controls, we stratified data by BMI, with a cut-off value of 25 kg/m ^2^. Subjects with BMI above 25 kg/m^2^ had 12% lower folate intake and 8.5% lower folate serum concentrations than did individuals with BMI below 25 kg/m^2^ (Table 3). However, despite the variations in folate intake and blood level, there were no significant differences in plasma Hcy concentrations between these subgroups (*p* = 0.15).

**Table 3:** Differences in selected parameters between normal weight people (BMI < 25) and overweight or obese people (BMI ≥ 25) aged 20–40 years

	BMI < 25	BMI ≥ 25	*P (t Student’s test)*
Parameters	Mean ± SD (95% CI)	Mean ± SD (95% CI)	
Plasma Hcy concentration [μmol/L]	10.49 ± 3.25 (10.0–10.9)	10.9 ± 3.34 (10.5–11.4)	NS
Serum folate concentration [ng/ml]	38.11 ± 12.9 (36.3–39.9)	34.9 ± 11.9 (33.3–36.5)	**< 0.01**
DFE intake [μg/day]	360 ± 200 (333–388)	318 ± 189 (292–344)	**< 0.05**

*P* values < 0.05 were considered significant

*Abbreviations*: *BMI* Body mass index, *DFE* Dietary folate equivalent, *Hcy* homocysteine, *NS* Not significant at *p* > 0.05

**Fig. 2:**
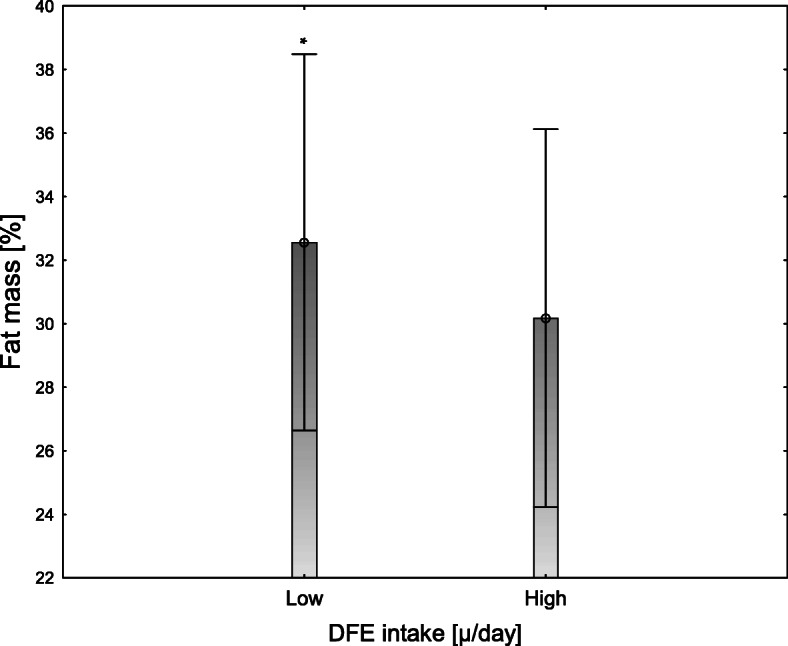
Body fat percentage stratified by DFE intake [ug/day], *n* = 421. Data as shown as means with their 95% confidence intervals. Low and high folate intake means below and above the median value, respectively. *Significantly different, *P* < 0.05

**Fig. 3:**
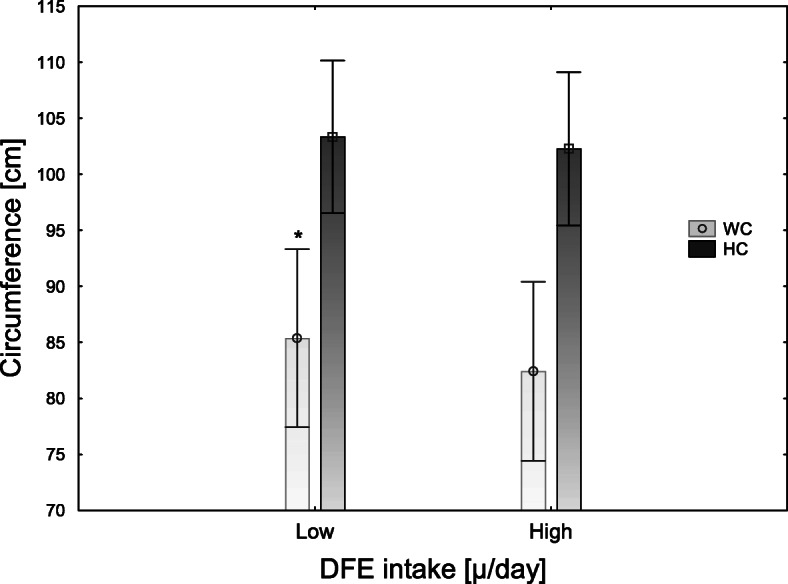
Waist and hip circumferences stratified by DFE intake [ug/day], *n* = 421. Data as shown as means with their 95% confidence intervals. Low and high folate intake means below and above the median value, respectively. *Significantly different, *P* < 0.05. Abbreviations: WC, waist circumference; HC, hip circumference

We examined the associations between folate metabolism and anthropometric measurements using regression models. As shown in Table 4*,* serum folate concentrations and folate intake were inversely associated with body fat percentage (*p* < 0.05) and waist circumference (*p* < 0.05 and *p* < 0.001 respectively); see Fig. 2 and Fig. 3. Furthermore, there was a negative association between serum folate concentration and WHR (*p* < 0.05), though the size of the effect of folate intake or concentration on the anthropometric variables was very low. Folate intake or folate serum concentration were not associated with hip circumference (Fig. 3). Furthermore, there were no associations between Hcy concentrations and any of the anthropometric measurements. We used a logistic regression model to analyze the associations between folate intake and status and BMI**.** Serum folate concentration, but not folate intake, was associated with BMI (*p* < 0.05); see Table 5. Moreover, we did not observe any associations between folate intake, serum folate, and Hcy concentrations.

**Table 4:** Associations between anthropometric parameters and folate intake, folate serum concentrations, and genes involved in folate metabolism in people aged 20–40 years; *n* = 421

	FM [%]			Waist circumference [m]			Hip circumference [m]			WHR		
	ß	partial η2	***p***	ß	partial η2	***p***	ß	partial η2	***p***	ß	partial η2	***p***
Sex, male	**−0.50**	**0.205**	**< 0.001**	**0.24**	**0.035**	**< 0.001**	**−0.26**	**0.047**	**< 0.001**	**0.21**	0.030	**< 0.001**
Low DFE intake [μg/day]	**0.11**	**0.014**	**< 0.05**	**0.11**	0.012	**< 0.05**	0.38	0.002	NS	0.06	0.002	NS
Serum folate level [ng/ml]	**−0.09**	**0.012**	**< 0.05**	**− 0.17**	**0.042**	**< 0.001**	− 0.06	0.003	NS	**−0.13**	0.061	**< 0.05**
*DHFR*, major allele	0.02	0.001	NS	0.01	0.012	NS	0.15	0.005	NS	−0.03	0.001	NS
*MTHFR*, major allele	−0.02	0.001	NS	−0.00	0	NS	0.65	0.000	NS	0.04	0.001	NS

*p* values < 0.05 were considered significant

*Abbreviations*: *FM* Fat mass, *NS* Not significant, *WHR* Waist-hip ratio

The regression models were adjusted for total energy intake and diet quality (as continuous), and misreporting and physical activity (as categorical)

**Table 5:** The odds ratio of being overweight or obese, *n* = 421

	OR (95% CI)	***p***
Sex, male	0.85 (0.55–1.30)	NS
DFE intake [μg/day]	0.98 (0.62–1.54)	NS
Serum folate concentration [ng/ml]	**0.98 (0.96–0.99)**	**< 0.05**
HDI	0.95 (0.81–1.11)	NS
Misreporting	**0.27 (0.16–0.46)**	**< 0.001**
Physical activity	0.78 (0.56–1.30)	NS

*p* values < 0.05 were considered significant

*Abbreviations*: *BMI* Body mass index, *CI* Confidence interval, *HDI* Healthy Diet Indicator, *NS* not significant at *p* > 0.05, *OR* odds ratio

Associations between BMI and folate intake and serum folate concentrations was calculated using logistic regression. The model was adjusted for total energy intake. Serum folate concentration was treated as continuous

## Discussion

In this study, we showed that low serum folate concentrations and low folate intakes are associated with greater BMI, higher body fat accumulation, and greater waist circumference, independently of the overall diet quality. Altogether, our findings support the hypothesis that higher folate intake could be a protective factor against obesity. An important strength of the study is that it is the first to consider folate intake, *MTHFR* and *DHFR* genotypes, serum folate levels, and plasma Hcy levels, controls for total energy intake, physical activity, and diet quality. The associations between BMI, as well as fat mass and folate concentration, have been presented in several studies before, in different age categories and populations (e.g. [14, 15, 22–26]). However none of these studies included as many confounding factors as here, especially intake of diet quality, which may have had a significant impact on the results. When the diet quality is not assessed, an effect assigned to folate intake may turn out to be an effect of the overall higher nutritional diet quality. It has previously been reported that starting with dietary pattern, then including nutrients, provides more reliable research outcomes, as it makes it possible to control for the effect of diet [27]. Furthermore, only a few studies have shown that folate serum concentration is lower in people with obesity and overweight, independently of folate intake (e. g. [15, 25, 26]).

The associations observed in this study could be explained in two ways: Firstly, low serum folate level may be an outcome of obesity, which could alter the pharmacokinetics of folate and lead to increased dietary folate requirements. Secondly, low serum folate level may be a cause of obesity through altered patterns of epigenetic modifications involved, for instance, in lipid metabolism [24]. Our finding that subjects with BMI > 25 kg/ m^2^ had lower serum folate independently of intake supports the hypothesis of da Silva et al. [18] that obesity may be an independent factor that alters folate redistribution by increasing the cellular uptake of dietary folate. Tinker et al. [23] have previously shown higher levels of RBC folate and lower serum folate in women with obesity, suggesting that people with obesity may store more folate in RBCs. However, the mechanism of such altered cellular uptake of folate remains unknown, and we did not measure RBC folate to analyze associations with anthropometrics. Another hypothesis was proposed by Bird et al. [28]—namely, that adiposity may affect the absorption of folate by the intestinal epithelium, which would be a second explanation of our findings. On the other hand, the study of da Silva et al. [18] denied this explanation, by showing that there were no differences in the area under the curve of serum folate concentration after an oral dose of 400 μg folic acid in women with obesity and in nonobese women aged 18–35; this suggested that absorption of folic acid is equal in all women. It should also be considered that obesity is often related to specific dietary patterns, which could be another factor affecting body weight. However, as mentioned above, the statistical model in our study included differences in diet quality, as well as differences in micronutrient and macronutrient intake models, to avoid confounding.

We also showed there to be associations between serum folate level and *MTHFR* genotype. Subjects with CT or TT genotype had lower serum folate levels than those with CC genotype. Despite the effect of genotype on folate levels, we did not find any associations between *MTHFR* polymorphism and anthropometric parameters. The l*ack of a* direct association may be a result of a very small effect of the *MTHFR* genotype on body weight status and composition; for this reason, although the study used 400 subjects, this sample size may not have been large enough to detect such an effect. Previous results for the associations between MTHFR C677T genotype and obesity are contradictory, and raise further questions. For instance, the study of Lewis et al. [29] showed that *MTHFR* C677T TT genotype was not associated with BMI, waist circumference, or WHR in two adult cohorts, but was associated with the prevalence of obesity. The study was performed on three large cohorts: The British Women’s Heart and Health Study (BWHHS), *n* = 3416; the Avon Longitudinal Study of Parents and Children (ALSPAC), *n* = 5130; and the Copenhagen City Heart Study (CCHS), *n* = 9173. In the ALSPAC cohort, the CT and TT genotypes were associated with lower BMI values in boys aged around nine. Contradicting these results, the same researchers found in the BWHHS cohort a positive association between TT genotype and the prevalence of obesity [29].

Another result of ours that deserves attention is the demonstration of an association between serum folate level, folate intake, and waist circumference, with a simultaneous lack of this association with hip circumference. This suggests that low folate status may cause only abdominal fat accumulation. These results are in line with the findings of Bird et al. [28] and of Piyathilak et al. [30]. The mechanisms linking folate deficiency and abdominal visceral adipose tissue accumulation could include systemic oxidative stress [31], glucose metabolism [26], and DNA methylation [32]. For instance, the study of Piyathilak et al. [30] showed that women with low plasma folate concentrations had lower PBMC (peripheral blood mononuclear cells) L1 methylation, which was related to higher waist circumference, BMI, and body fat percentage. This finding suggests that folate affects body fat distribution through epigenetic mechanisms.

According to our results and those of others [15], lower serum folate concentrations in subjects with overweight and obesity are unrelated to Hcy concentration. We have observed a lack of association between folate and Hcy level, in accordance with Fonseca et al. [33], as well as between Hcy level and anthropometric measurements. Our results are in line with those of Fonseca et al. and Terruzzi et al. [33, 34], where no relationships were found between Hcy levels and BMI. Further, we did not find an association between *MTHFR* genotype and Hcy level, but Hcy metabolism depends on many factors, including metabolic capacity, diet, gender and, age. Some previous studies have reported different findings. In contrast to our study, Pereira et al. [16] reported that serum folate levels are positively associated with dietary folate and negatively associated with serum Hcy. Yet a few studies have also indicated Hcy to be positively correlated with BMI, fat mass, and waist circumference [35, 36], which suggests that Hcy may not be directly involved in mechanisms leading to obesity, but may in some cases act as an indicator of metabolic abnormalities.

This study has several limitations. First, we focused only on short-term folate status and did not measure RBC folate; we thus did not have a comprehensive view of folate status. Second, daily dietary folate intake was calculated as a mean value of 3 days’ intake using three-day food records, which may be too short a period to mirror daily folate intake. We only studied people aged 20–40, so the conclusions cannot be generalized to other age groups.

Taken together, our study showed folate is one of dietary micronutrients associated with increased body weight development, and so it may be reasonable to increase the daily recommendation of folate intake for people with obesity, who may have greater requirements for folate intake.

## Conclusion

We have demonstrated that low folate intake and serum level are weakly, but independently, associated with greater body weight and central adiposity in healthy adults aged 20–40. Moreover, there is a significant association between *MTHFR* genotype and serum folate level. However, *MTHFR* and *DHFR* polymorphism seems not to have significant impact on body weight.

## Data Availability

The datasets used and/or analysed during the current study are available from the corresponding author on reasonable request.

## Abbreviations

BMI: Body mass index
DFE: Dietary folate equivalent
DHFR: Dihydrofolate reductase
Hcy: Homocysteine
HDI: Healthy Diet Indicator
MTHFR: Methylene tetrahydrofolate reductase
PBMC: Peripheral blood mononuclear cells
RBC: Red blood cells
THF: Tetrahydrofolate
WHR: Waist to hip ratio
